# Identification and comparative analysis of differentially expressed miRNAs in leaves of two wheat (*Triticum aestivum* L.) genotypes during dehydration stress

**DOI:** 10.1186/s12870-015-0413-9

**Published:** 2015-01-27

**Authors:** Xingli Ma, Zeyu Xin, Zhiqiang Wang, Qinghua Yang, Shulei Guo, Xiaoyang Guo, Liru Cao, Tongbao Lin

**Affiliations:** College of Agronomy, Henan Agricultural University, Zhengzhou, 450002 China; Collaborative Innovation Center of Henan Grain Crops, Zhengzhou, 450002 China; National Key Laboratory of Wheat and Maize Crop Science, Zhengzhou, 450002 China

**Keywords:** Dehydration stress, *Triticum aestivum* L, Differentially expressed miRNAs, Comparative analysis

## Abstract

**Background:**

MicroRNAs (miRNAs) play critical roles in the processes of plant growth and development, but little is known of their functions during dehydration stress in wheat. Moreover, the mechanisms by which miRNAs confer different levels of dehydration stress tolerance in different wheat genotypes are unclear.

**Results:**

We examined miRNA expressions in two different wheat genotypes, Hanxuan10, which is drought-tolerant, and Zhengyin1, which is drought-susceptible. Using a deep-sequencing method, we identified 367 differentially expressed miRNAs (including 46 conserved miRNAs and 321 novel miRNAs) and compared their expression levels in the two genotypes. Among them, 233 miRNAs were upregulated and 10 were downregulated in both wheat genotypes after dehydration stress. Interestingly, 13 miRNAs exhibited opposite patterns of expression in the two wheat genotypes, downregulation in the drought-tolerant cultivar and upregulation in the drought-susceptible cultivar. We also identified 111 miRNAs that were expressed predominantly in only one or the other genotype after dehydration stress. We verified the expression patterns of a number of representative miRNAs using qPCR analysis and northern blot, which produced results consistent with those of the deep-sequencing method. Moreover, monitoring the expression levels of 10 target genes by qPCR analysis revealed negative correlations with the levels of their corresponding miRNAs.

**Conclusions:**

These results indicate that differentially expressed patterns of miRNAs between these two genotypes may play important roles in dehydration stress tolerance in wheat and may be a key factor in determining the levels of stress tolerance in different wheat genotypes.

**Electronic supplementary material:**

The online version of this article (doi:10.1186/s12870-015-0413-9) contains supplementary material, which is available to authorized users.

## Background

Drought is a major environmental stress factor worldwide that affects plant growth and development. Under drought stress, a series of protective mechanisms are triggered that allow plants to adapt to adverse conditions [1,2]. Phytohormones and second-messenger molecules participate in signal transduction to respond to stress by inducing expression of both protein-coding and non-protein-coding genes to produce regulatory molecules, effector molecules directly involved in the biochemical response, and products of non-protein coding genes that regulate expression of other genes at the transcriptional and translational levels [1,3].

As non-protein-coding gene products, microRNAs (miRNAs), ranging in length from 18 to 25 nucleotides, regulate gene expression either through post-transcriptional degradation or translational repression of their target mRNAs. In plants, most miRNAs have perfect or near-perfect complementarity to their mRNA targets and downregulate them by targeted cleavage or translational repression [4,5]. Functional analyses have demonstrated that miRNAs are involved in a variety of developmental processes in plants [6]. For instance, miR156, miR166, miR168 and miR2009 show abundant expression in young wheat seedlings [7]. Recently, 323 wheat novel miRNAs are characterized in a genome-wide level and further identified 64 miRNAs preferentially expressing in developing or germinating grains, which could play important roles in grain development [8]. In addition, miRNAs play critical roles in plant resistance to various abiotic and biotic stresses [9-11]. For example, in the thermosensitive genic male sterile (TGMS) lines of wheat, miR167, miR172, miR393, miR396 and miR444c.1 are found to respond to cold stress. Interestingly, miR167 play roles in regulating the auxin-signaling pathway and possibly in the developmental response to cold stress [12]. Similarly, the expression levels of miR156, miR159, miR164, miR167a, miR171, miR395 and miR6000 have been shown to be altered in wheat under UV-B stress [13]. Besides, miR827 and miR2005 are up-regulated in wheat both under powdery mildew infection and heat stress, whereas miR156, miR159, miR168, miR393, miR2001, and miR2013 exhibit opposite expression pattern response to these stresses [14].

miRNA expression profiling after drought stress has been performed in wild emmer wheat, rice, *Arabidopsis* and *Populus.* Previously, miR1867, miR474, miR398, miR1450, miR1881, miR894, miR156, and miR1432 have been found to be induced by drought in wild emmer wheat (*Triticum dicoccoides*) [3]. Similarly, miR169g is strongly induced while miR393 is transiently upregulated in rice by drought stress [15]. Several miRNAs (miR156, miR159, miR168, miR170, miR172, miR319, miR396, miR397, miR408, miR529, miR896, miR1030, miR1035, miR1050, miR1088, and miR1126) are found to be downregulated and 14 miRNAs (miR159, miR169, miR171, miR319, miR395, miR474, miR845, miR851, miR854, miR896, miR901, miR903, miR1026, and miR1125) are revealed to be induced by drought stress in rice [16]. In *Arabidopsis*, miR167, miR168, miR171, and miR396 are shown to be drought responsive [17]. In *Populus*, miR171l-n, miR1445, miR1446a-e, and miR1447 also have been proved to respond to drought stress [18].

Although numerous miRNAs have been identified in many plant species, only 42 sequences have been reported for wheat in the miRBase registry (miRBase release 20). Furthermore, how miRNAs confer different levels of dehydration stress tolerance in various wheat genotypes is unclear. To gain insight into the role of wheat miRNAs in dehydration stress tolerance, two representative wheat genotypes were used in this study: Hanxuan10, a drought-tolerant cultivar grown widely in dry land wheat regions of North China; and Zhengyin1, which is drought-susceptible and often planted in water- and fertilizer-rich regions. We grew these two genotypes under well-watered and dehydration-stress conditions and analyzed miRNA expression patterns to identify those miRNAs involved in dehydration stress tolerance.

## Results

### Effects of dehydration stress on phenotypic alteration to two wheat genotypes

**Figure 1:**
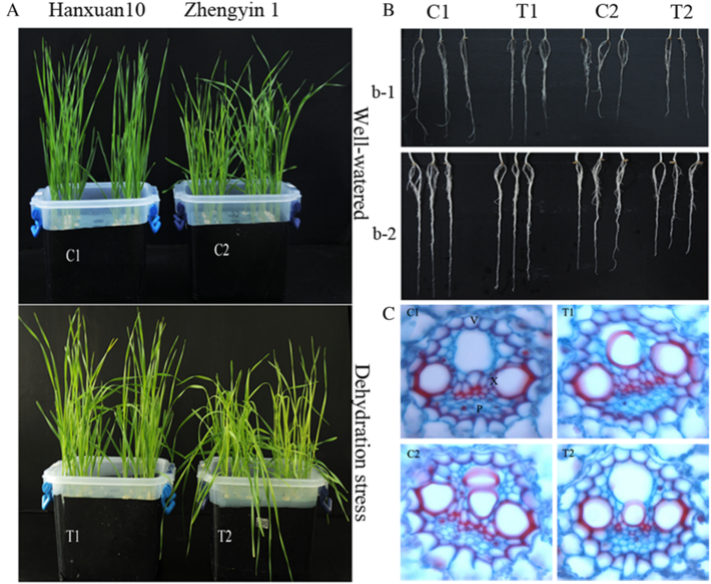
**Effects of dehydration stress on phenotypic alteration to wheat seedlings.** **(A)** Morphological changes in two wheat genotypes after 12h dehydration stress. **(B)** Effect of dehydration stress on growth and development of lateral roots of the two wheat genotypes. Changes in the numbers and length of lateral roots in two wheat genotypes after 12h (b-1) and 72h (b-2) dehydration treatment. **(C)** Effect of dehydration stress on differentiation of vascular tissue cells of leaves in the two wheat genotypes (×40). V, vascular bundle sheath; X, xylem; P, phloem.

The two wheat genotypes exhibited morphological differences after 12-h dehydration stress treatment. While the Hanxuan 10 plants (T1) continued to grow relatively well, the plants of Zhengyin 1 (T2) displayed severe dehydration stress symptoms, such as wilting leaves (Figure 1A). In addition, the chlorophyll content of T1 and T2 decreased by 12.87% and 16.73% than that of C1 and C2, and relative water content of T1 and T2 decreased by 4.70% and 10.58% after dehydration stress, respectively (Additional file 1: Table S1).

**Figure 2:**
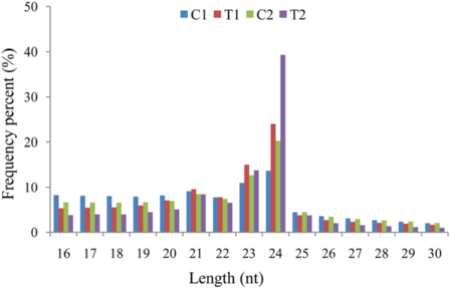
**Size distribution of wheat small RNAs.** C1 and C2 indicate well-watered Hanxuan10 (drought-tolerant cultivar) and Zhengyin1 (drought-susceptible cultivar). T1 and T2 indicate dehydration-stressed Hanxuan10 and Zhengyin1.

The growth and development of lateral roots showed obvious differences in two wheat genotypes after dehydration treatment (Figure 1B). For example, the total lengths of lateral roots of C1, T1, C2 and T2 were 68.74, 65.98, 50.72 and 47.54 cm after 12h dehydration stress, respectively (Figure 1b-1 and Table 1). By stress time increasing, the total lengths of lateral roots of T1 and T2 were 79.90 and 51.90 cm after 72h dehydration stress, whereas the total length of lateral root were 90.96 and 64.66 cm in their corresponding control (Figure 1b-2 and Table 1). Compared with the total lengths after 12h stress, the total lengths of lateral roots of C1, T1 and C2 increased respectively by 22.22, 13.92 and 13.94 cm, but T2 only increased by 4.36 cm. Moreover, numbers of lateral roots were also changed by dehydration stress. For instance, numbers of lateral roots of T2 decreased by 0.8 than C2 after 12h dehydration stress, but T1 only decreased by 0.2 than C1 (Table 1). These results suggested that dehydration stress significantly inhibited lateral roots growth and development of the drought-susceptible cultivar, but had a lesser effect on the drought-tolerant cultivar.

**Table 1:** **Changes in the numbers and length of lateral roots in two wheat genotypes after dehydration stress**

**Treatments**	**12h after stress**		**72h after stress**	
	**Numbers of lateral roots**	**Total length of lateral roots (cm)**	**Numbers of lateral roots**	**Total length of lateral roots (cm)**
C1	5.4 ± 0.55	68.74 ± 2.30	6.2 ± 0.45	90.96 ± 2.64
T1	5.2 ± 0.83	65.98 ± 2.61	5.4 ± 0.55*	79.90 ± 5.23
C2	5.0 ± 0.71	50.72 ± 4.34**	5.2 ± 0.84*	64.66 ± 3.93**
T2	4.2 ± 0.84*	47.54 ± 2.75**	4.2 ± 0.45**	51.90 ± 3.31**

The data are mean ± SD (n = 5). *,**Indicate significant difference at P < 0.05 and P < 0.01, respectively.

We found that the number of leaf vascular tissue cells in two wheat genotypes showed distinct differences after 12h dehydration stress (Figure 1C). For instance, xylem and phloem cells of T1 leaves were increased averagely by 2.7 and 0.6 compare with C1 after dehydration treatment, respectively. However, xylem and phloem cells of T2 were decreased by 9.0 and 8.0 compared to C2 after dehydration stress, respectively (Table 2). These results implied that dehydration stress suppressed dramatically differentiation of vascular tissue cells of leaves of the drought-susceptible cultivar, but differentiation was promoted in the drought-tolerant cultivar.

**Table 2:** **Changes in the numbers of vascular bundle sheath, xylem and phloem in two wheat genotypes after dehydration stress**

**Treatments**	**Numbers of vascular bundle sheath**	**Numbers of xylem cell**	**Numbers of phloem cell**
C1	20.3 ± 0.57**	37.3 ± 2.08*	35.7 ± 0.57**
T1	22.7 ± 0.57	40.0 ± 3.61	36.3 ± 0.33**
C2	19.7 ± 1.15**	42.0 ± 1.00	41.7 ± 2.03
T2	20.7 ± 0.57*	33.0 ± 1.00**	33.7 ± 0.33**

The data are mean ± SD (n = 3). *, **Indicate significant difference at P < 0.05 and P < 0.01, respectively.

### Sequencing and annotation of wheat miRNAs

Solexa sequencing of miRNA libraries generated from well-watered (C1) and dehydration-stressed (T1) Hanxuan10 and well-watered (C2) and dehydration-stressed Zhengyin1 (T2) plants yielded 20653733, 19546412, 19375732, and 21290140 unfiltered sequence reads, respectively. After discarding low-quality reads, a total of 12005904 (58.13%, C1), 10544528 (53.95%, T1), 10619535 (54.81%, C2), and 11701889 (54.96%, T2) reads were retained. These sequences represented 650391 (3.15%), 1046638 (5.35%), 846328 (4.37%), and 1798773 (8.45%) unique clean reads for C1, T1, C2, and T2, respectively (Table 3). The most abundant classes of these unique clean reads were 21–24 nucleotides (nt), and the 24 nucleotides (nt) sequences were the most common (Figure 2). The unique reads were compared sequentially with the Rfam and miRBase databases to annotate 228251, 253538, 253662, and 303835 unique small RNAs (sRNAs) and 1451 (0.64%), 1697 (0.67%), 1615(0.64%), and 2056 (0.68%) unique miRNAs for C1, T1, C2, and T2, respectively (Table 4).

**Table 3:** **Small RNA sequences present in C1, T1, C2 and T2 plants**

**Treatments**	**Total reads number**	**Clean number**	**Unique number**
	**(Percentage)**	**(Percentage)**	**(Percentage)**
C1	20653733(100%)	12005904(58.13%)	650391(3.15%)
T1	19546412(100%)	10544528(53.95%)	1046638(5.35%)
C2	19375732(100%)	10619535(54.81%)	846328(4.37%)
T2	21290140(100%)	11701889(54.96%)	1798773(8.45%)

**Table 4:** **Annotation of sRNAs sequences from C1, T1, C2 and T2**

**Category**	**Unique signatures**				**Total signatures**			
	**C1**	**T1**	**C2**	**T2**	**C1**	**T1**	**C2**	**T2**
rRNA	112291(49.20%)	126346(49.83%)	133724(52.72%)	147784(48.64%)	1995335(27.20%)	2054201(34.83%)	2095372(28.60%)	2517033(40.42%)
tRNA	37394(16.38%)	37971(14.98%)	35593(14.03%)	45462(14.96%)	3764841(51.32%)	2166029(36.73%)	4133757(56.43%)	2557011(41.06%)
snoRNA	17488(7.66%)	20634(8.14%)	18452(7.27%)	24474(8.06%)	850682(11.59%)	893967(15.16%)	295169(4.03%)	141849(2.29%)
snRNA	9485(4.16%)	11277(4.45%)	10164(4.01%)	13596(4.47%)	60773(0.83%)	64856(1.10%)	47312(0.65%)	59258(0.95%)
miRNA	1451(0.64%)	1697(0.67%)	1615(0.64%)	2056(0.68%)	109924(1.50%)	268389(4.55%)	80626(1.10%)	407419(6.54%)
Other	50142(21.97%)	55613(21.93%)	54114(21.33%)	70463(23.19%)	554968(7.56%)	449981(7.63%)	673621(9.19%)	544438(8.74%)
Total	228251(100%)	253538(100%)	253662(100%)	303835(100%)	7336523(100%)	5897423(100%)	7325857(100%)	6227008(100%)

### Comparison of differentially expressed miRNAs between two wheat genotypes

We compared the frequencies of occurrence of differentially expressed miRNAs in well-watered and dehydration-stressed plants based on a Poisson distribution approach [19]. We identified 71 conserved miRNAs from Hanxuan10 and 102 conserved miRNAs from Zhengyin1 that were differentially expressed between well-watered and dehydration-stressed treatment (Additional file 2: Tables S2-3 and S2-4). We focused on those miRNAs common to Hanxuan10 and Zhengyin1 and compared their expression levels after dehydration treatment. We used the following criteria as the basis for comparison: a log2 ratio of normalized values between the dehydration stress and control treatments greater than 1 or less than −1 in one of the two genotypes. We identified 46 miRNAs in common between the two wheat genotypes that were differentially expressed in response to the dehydration treatment (Additional file 2: Table S2-5). Through comparative analysis, we observed that 14 miRNAs showed upregulation in both genotypes after dehydration stress (Table 5), while another 6 miRNAs were downregulated (Table 6). The expression of 13 miRNAs exhibited opposite patterns in the two wheat genotypes (Table 7); these miRNAs were downregulated in Hanxuan10 but upregulated in Zhengyin1. In addition, 13 miRNAs were expressed predominantly in only one or the other of the two genotypes after dehydration-stress treatment (Table 8).

**Table 5:** **Upregulated miRNAs in both two wheat genotypes after dehydration stress**

**miRNAs ID**	**Homologous miRNAs**	**Normalized value (TPM)**				**Log2**	**Log2**	**Putative target**
		**C1**	**T1**	**C2**	**T2**	**(T1/C1)**	**(T2/C2)**	
tae-miR156k	gma-miR156k	0.25	1.71	0.19	5194.72	2.77	14.75	SBP
tae-miR159a-5p	gma-miR159a-5p	0.17	1.61	1.04	133.40	3.27	7.01	Serine/arginine repetitive matrix 1
tae-miR166l-5p	osa-miR166l-5p	0.92	33.48	0.01	1.62	5.19	7.34	FAM10 family protein
tae-miR166n-5p	osa-miR166n-5p	0.58	38.03	0.01	1.28	6.03	7.00	
tae-miR167b	sof-miR167b	0.42	15.74	1.60	233.55	5.24	7.19	
tae-miR168a-5p	zma-miR168a-5p	5.75	556.21	1.79	46.92	6.60	4.71	
tae-miR168b	sof-miR168b	3.66	16.60	2.45	220.22	2.18	6.49	Short-chain dehydrogenase/reductase
tae-miR444c.1	osa-miR444c.1	4.83	23.99	38.7	98.02	2.31	1.34	MADS-box transcription factor
tae-miR827b	osa-miR827b	0.25	0.76	0.28	204.50	1.60	9.50	ATP-dependent Clp protease
tae-miR829-3p	aly-miR829-3p	0.01	0.19	0.19	15.21	4.25	6.34	Purple acid phosphatase-like protein
tae-miR1137	tae-miR1137	18.16	72.36	33.99	170.23	1.99	2.32	Pherophorin-C1 protein precursor
tae-miR1318-3p	osa-miR1318-3p	1.17	2.56	11.02	35.64	1.13	1.69	
tae-miR1432	osa-miR1432	10.16	32.34	0.56	16.32	1.67	4.85	Mitochondrial phosphate transporter
tae-miR5368	gma-miR5368	1671.09	3890.36	580.25	2146.66	1.22	1.89

**Table 6:** **Downregulated miRNAs in both two wheat genotypes after dehydration stress**

**miRNAs ID**	**Homologous miRNAs**	**Normalized value (TPM)**				**Log2**	**Log2**	**Putative target**
		**C1**	**T1**	**C2**	**T2**	**(T1/C1)**	**(T2/C2)**	
tae-miR159a	ath-miR159a	1036.32	4.08	19.40	1.28	−7.99	−3.92	MYB3
tae-miR159b	mdm-miR159b	0.17	0.01	2.45	0.17	−4.06	−3.84	MYB3
tae-miR159c-5p	aly-miR159c-5p	36.73	4.27	18.64	6.92	−3.11	−1.43	Dihydro-flavanoid reductase-like protein
tae-miR171f	sbi-miR171f	3.00	0.19	2.45	0.26	−3.98	−3.26	Sensor histidine kinase
tae-miR395i	osa-miR395i	0.75	0.19	7.25	1.88	−1.98	−1.95	ATP sulfurylase
tae-miR916	cre-miR916	18.49	7.97	21.19	7.95	−1.21	−1.41

**Table 7:** **Opposite expression miRNAs in both two wheat genotypes after dehydration stress**

**miRNAs ID**	**Homologous miRNAs**	**Normalized value (TPM)**				**Log2**	**Log2**	**Putative target**
		**C1**	**T1**	**C2**	**T2**	**(T1/C1)**	**(T2/C2)**	
tae-miR160a	vvi-miR160a	5.16	0.19	0.19	13.33	−4.77	6.15	ARF
tae-miR164b	sbi-miR164b	15.24	0.01	0.19	1.37	−10.57	2.86	NAC
tae-miR166h	cme-miR166h	5.83	0.85	0.38	0.94	−2.77	1.32	HD-ZIP4
tae-miR169d	vvi-miR169d	4.50	0.28	0.19	5.73	−3.98	4.93	CCAAT-box transcription factor
tae-miR172a	bdi-miR172a	7.66	2.75	0.28	2.31	−1.48	3.03	Succinyl CoA ligase beta subunit-like protein
tae-miR319c	ppt-miR319c	6.41	0.38	0.19	1.20	−4.08	2.67	Acyl-CoA synthetase
tae-miR393b	mdm-miR393b	8.08	1.04	1.22	410.19	−2.95	8.39	TIR1
tae-miR393i	gma-miR393i	9.58	1.99	0.19	26.49	−2.27	7.14	TIR1
tae-miR396a	bdi-miR396a	129.69	29.11	12.62	332.6	−2.16	4.72	GRF
tae-miR396c	zma-miR396c	8169.4	67.24	6.4	43.84	−6.92	2.78	GRF
tae-miR396g	osa-miR396g	13.99	3.13	0.47	16.83	−2.16	5.16	GRF
tae-miR444d.3	osa-miR444d.3	5.83	0.38	0.01	0.17	−3.94	4.09	IF3
tae-miR827-5p	zma-miR827-5p	28.57	0.01	0.19	0.85	−11.48	2.18	PHD finger-like protein

**Table 8:** **Differentially expressed miRNAs only in one wheat genotype after dehydration stress**

**miRNAs ID**	**Homologous miRNAs**	**Normalized value (TPM)**				**Log2**	**Log2**	**Putative target**
		**C1**	**T1**	**C2**	**T2**	**(T1/C1)**	**(T2/C2)**	
tae-miR156h	mdm-miR156h	0.42	0.47	0.56	89.56	0.19	7.31	SBP
tae-miR159a.2	osa-miR159a.2	5.00	2.75	0.47	1538.47	−0.86	11.67	Ent-kaurene synthase
tae-miR319a-3p	osa-miR319a-3p	8.33	6.16	7.91	3.85	−0.43	−1.04	Probable dihydrodipicolinate reductase 1
tae-miR398	tae-miR398	1.67	3.03	53.11	453.43	0.87	3.09	Superoxide dismutase[Cu-Zn]
tae-miR528b-3p	zma-miR528b-3p	0.33	0.19	0.38	187.23	−0.81	8.96	Receptor protein kinase-like
tae-miR538a	ppt-miR538a	52.22	5.78	8.57	6.15	−3.17	−0.48	
tae-miR1128	ssp-miR1128	0.67	0.76	2.17	0.85	0.19	−1.34	Irvingia malayana 18S ribosomal RNA gene
tae-miR1310	pta-miR1310	73.38	75.96	98.40	35.21	0.05	−1.48	
tae-miR1862b	osa-miR1862b	1.58	0.85	4.71	0.85	−0.89	−2.46	Myosin heavy chain class VIII A2 protein
tae-miR2911	peu-miR2911	343.66	641.75	322.24	864.82	0.90	1.42	Chlorophyll a/b-binding protein WCAB precursor
tae-miR5048b	hvu-miR5048b	116.53	736.59	170.82	312.09	2.66	0.87	Protein kinase domain containing protein
tae-miR5059	bdi-miR5059	7.75	7.11	17.99	7.78	−0.12	−1.21	
tae-miR5648-5p	ath-miR5648-5p	6.75	2.56	1.51	0.77	−1.40	−0.97	Aquaporin NIP1-2

In addition,to identify the novel miRNAs, criteria for annotation of plant miRNAs [20] were used in our study. Finally, 521 novel miRNAs were predicted based on the hexaploid wheat genome (http://www.cerealsdb.uk.net/CerealsDB/Documents/DOC_CerealsDB.php). According to the screening criteria of differentially expressed miRNAs, we found that 321 novel miRNAs were differentially expressed in two wheat genotypes after dehydration stress (Additional file 3: Table S3). Among them, 219 miRNAs showed upregulation in both genotypes after dehydration stress, while another 4 miRNAs were downregulated. Moreover, 98 miRNAs were expressed predominantly in only one of the two wheat genotypes after dehydration stress (Additional file 3: Tables S3-2, S3-3 and S3-4).

### Validation of differentially expressed miRNAs

**Figure 3:**
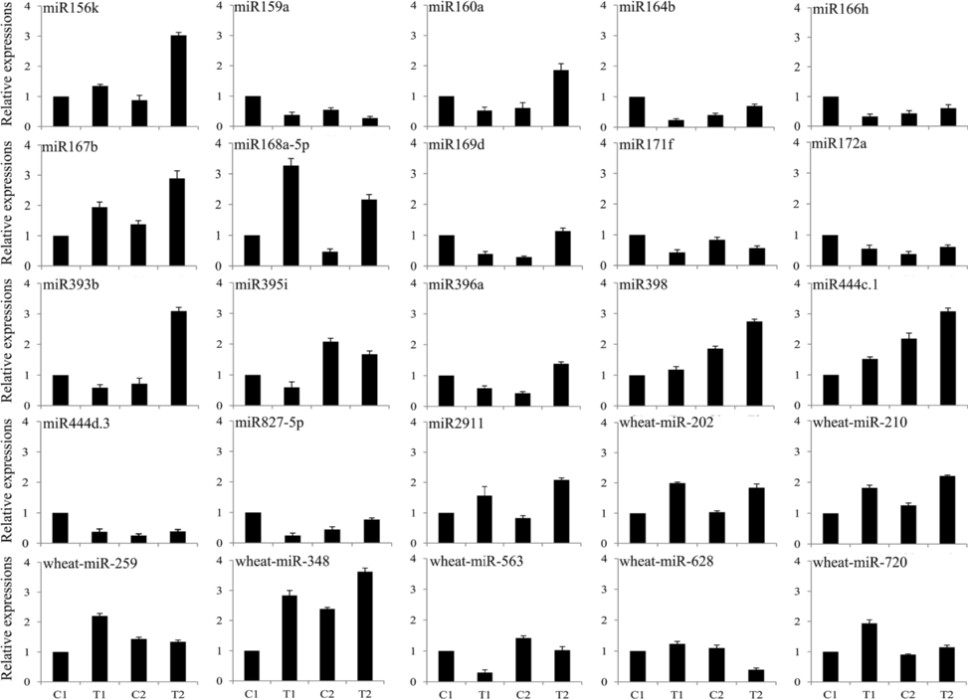
**Comparison of the expression levels of 25 miRNAs in two wheat genotypes.** miRNA copy numbers were normalized by comparison with wheat 18S rRNA; individual miRNA expression levels were then normalized by comparison with their expression in the C1 well-watered control treatment, which was set to 1.0. The experiments were repeated three times and error bars represent standard deviations.

**Figure 4:**
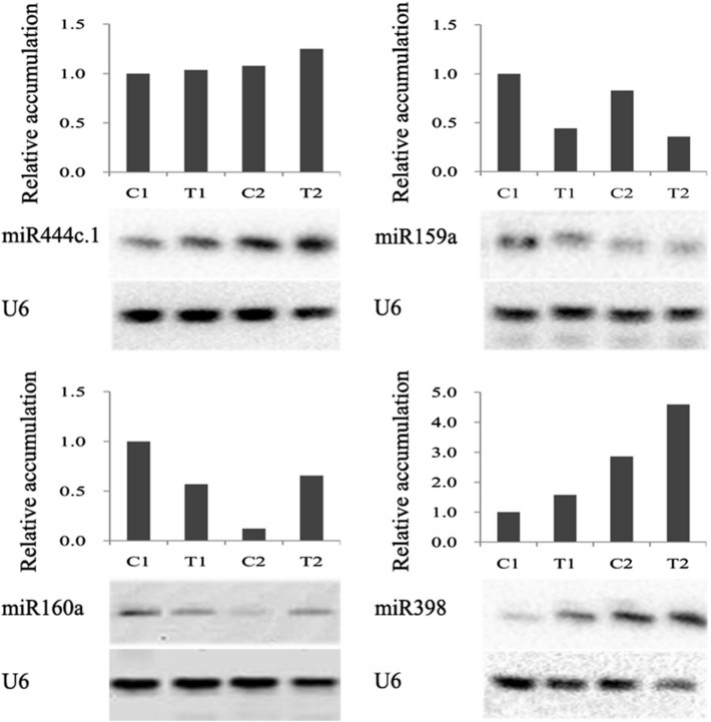
**Northern blot analysis of the expression of 4 miRNAs in two wheat genotypes after 12h dehydration stress.** U6 was used as a loading control. The relative accumulation levels of miRNA to U6 are shown in histograms. The levels of each miRNA were normalized by comparison with their expression in the C1 well-watered control treatment, which was set to 1.0.

To confirm the results of the deep sequencing and comparative analyses, we verified the expression patterns of 25 miRNAs selected randomly by qPCR. The qPCR results coincided with those of the deep sequencing (Figure 3). For example, miR160a, miR164b, miR166h, miR169d, and miR444d.3 were confirmed by both techniques to be downregulated in the drought-tolerant Hanxuan10 after dehydration stress but upregulated in the drought-susceptible Zhengyin1 (Table 7 and Figure 3). Similarly, miR156k, miR444c.1 and wheat-miR-202 (a novel miRNA, secondary structure shown in Additional file 4: Table S4) were shown by both methods to be upregulated in both wheat genotypes after dehydration stress (Table 5, Additional file 3: Table S3-2 and Figure 3), miR398 and wheat-miR-628 (a novel miRNA) were expressed predominantly in only one of the two genotypes (Table 8, Additional file 3: Table S3-4 and Figure 3). Northern blot was also performed to study the transcripts of miRNAs of four different expression patterns to confirm the expression profiles obtained from deep sequencing (Figure 4). The results showed that expression of these miRNAs in different treatments was also consistent with the result of high-throughput sequencing. These results indicated that the frequency of occurrence in the Solexa runs produced a reliable prediction of expression patterns.

### Prediction and validation of miRNA functions and their effects on potential targets

**Figure 5:**
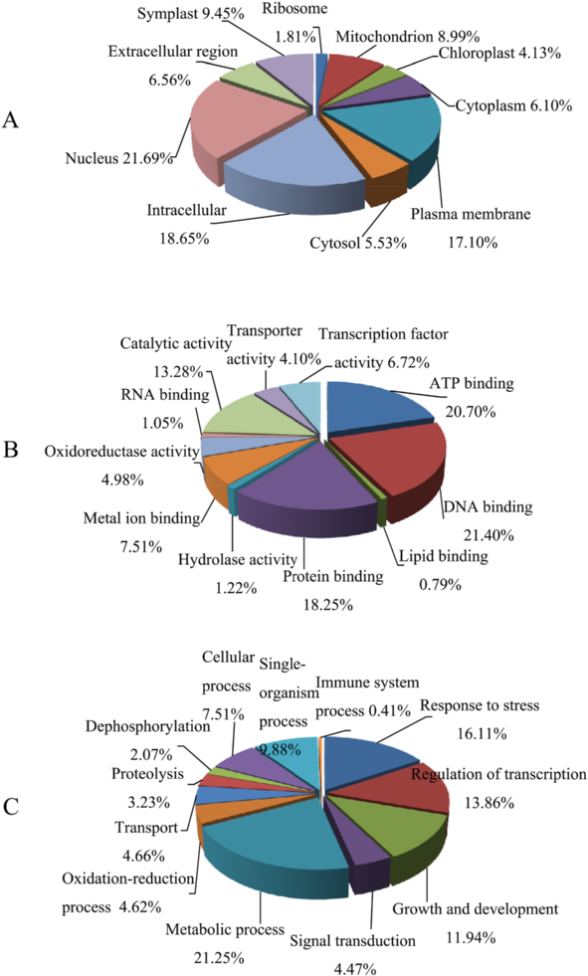
**Gene ontology of the predicted target genes of 367 differentially expressed miRNAs.** Categorization of miRNA-target genes was performed according to the cellular component **(A)**, molecular function **(B)**, and biological process **(C)** categories.

**Figure 6:**
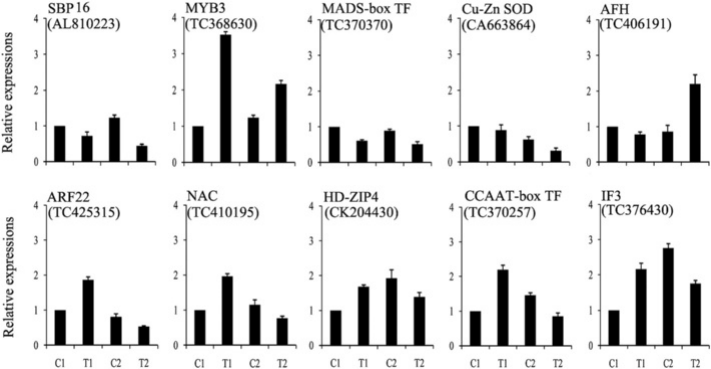
**Comparison of expression levels of 10 target genes in two wheat genotypes.** The copy numbers of target mRNAs were normalized by comparison with wheat 18S rRNA; expression levels of each target gene were then normalized by comparison with their expression in the C1 well-watered control treatment, which was set to 1.0. The experiments were repeated three times and error bars represent standard deviations. SBP16, squamosa promoter-binding-like protein 16; MYB3, MYB3 transcription factor; MADS-box TF, MIKC-type MADS-box transcription factor; Cu-Zn SOD, Cu-Zn superoxide dismutase; AFH, alpha/beta fold hydrolase; ARF22, auxin response factor 22; NAC, NAC transcription factor; HD-ZIP4, Class III HD-ZIP protein 4; CCAAT-box TF, CCAAT-box transcription factor; IF3, translation initiation factor 3.

We predicted 1805 target genes for the 367 differentially expressed miRNAs (including 46 conserved miRNAs and 321 novel miRNAs, Additional file 5: Tables S5-1 and S5-2). These potential targets were assigned based on Gene Ontology. With respect to molecular function, the targets fell largely into 11 categories, with the three most over-represented being DNA binding, ATP binding, and protein binding. Twelve biological processes were identified, with the three most frequent being metabolic process, response to stress, and regulation of transcription (Figure 5). Furthermore, monitoring the expression levels of 10 representative target genes by qPCR analysis revealed negative correlations with the levels of their corresponding miRNAs (Figure 6). These results implied that several miRNAs may be directly or indirectly involved in wheat tolerance to dehydration stress through regulation of target gene expression.

## Discussion

Recent studies have indicated that the expression of miRNAs, an important class of gene regulators, is altered by abiotic stress treatment [21-23]. However, most of these studies were performed using model organisms such as Arabidopsis and rice. In this work, we investigated changes in miRNA expression levels after dehydration stress in two wheat genotypes to better understand the function of plant miRNAs in stress adaptation.

In this study, we identified 14 upregulated conserved miRNAs and 6 conserved downregulated miRNAs (Tables 5 and 6) in two wheat genotypes subjected to dehydration stress. The gene target of the upregulated miR156k encodes the squamosa promoter-binding-like protein (SBP) transcription factor, which is known to be important for leaf growth and development [24]. The target of the upregulated miR444c.1 is the MIKC-type MADS-box transcription factor (MADS-box TF) gene, which was reported to be involved in regulating plant developmental processes and stress responses [25]. For the downregulated miR159a, the gene target encodes the MYB3 transcription factor, which plays a role in cold-stress responses [26]. MYB family members have also been implicated in plant tolerance to environmental stress through their functions in hormone and other abiotic stress signaling networks [27]. Our findings indicate that these miRNAs may also play important roles in stress tolerance in wheat.

Genotypic specificity of miRNA expression has been reported previously in terms of the differential expression of a given miRNA in the same tissues in different genotypes [28]. In this study, we found that 13 conserved miRNAs and 98 novel miRNAs were expressed predominantly in only one or the other genotype after dehydration treatment (Table 8 and Additional file 3: Table S3-4). For example, miR398 was upregulated in the drought-susceptible cultivar after dehydration treatment (Table 8 and Figure 3). This miRNA has been reported to be upregulated in response to copper deprivation [29] and its target gene, superoxide dismutase, is induced during oxidative stress [30,31]. We also showed that wheat-miR-628 (a novel miRNA) was downregulated only in the drought-susceptible cultivar (Additional file 3: Table S3-4 and Figure 3) and its putative gene target was alpha/beta fold hydrolase (AFH). Most hydrolases are believed to be involved in the decomposition of products of damage (‘cell cleaning’) caused by stress conditions [32]. Moreover, AFHs may have diverse functions and play various roles in different pathways despite their sequence similarities. In some cases, they may function as enzymes such as proteases, esterases, or peroxidases [33]. Our findings suggest that the different expression patterns of wheat-miR-628 among wheat genotypes may be related to variations in the capacity to adapt to dehydration stress.

A different expression pattern was exhibited by 13 miRNAs that were downregulated in the drought-tolerant cultivar, but were upregulated in the drought-susceptible cultivar including miR160a, miR164b, miR166h, miR169d, and miR444d.3 (Table 7 and Figure 3). The putative target of miR160a is a member of the auxin response factors (ARFs) gene family. ARFs are key factors in the regulation of physiological and morphological mechanisms mediated by auxins that may contribute to stress adaptation [34]. Furthermore, ARFs regulate the expression of early auxin responsive genes, including the AUX/IAA genes [35], and AUX/IAA proteins interact with ARFs and repress their activities [36]. Auxin induces targeted ubiquitination/degradation of specific AUX/IAA proteins [37] and frees ARFs from repression by AUX/IAA proteins. The accumulation of ARFs resulting from the downregulation of miR160a might enhance the auxin response and thus enhance root and leaf development. The target of miR164b is the NAC transcription factor (NAC TF) family. NAC TFs have functions related to various abiotic stress [38,39]; indeed, overexpression of the SNAC1 gene in rice increased drought and salt tolerance [40]. In *Arabidopsis*, NAC1 overexpressing lines were bigger, with larger leaves, thicker stems and more abundant roots than their control plants. The NAC1 might be an early auxin responsive gene, and confirmed that NAC1 was located downstream of TIR1 and upstream of AIR3 and DBP in transmitting the auxin signal to the AIR3 gene to promote lateral root’s development. TIR1 is likely to regulate NAC1 at the transcriptional level, perhaps through auxin-dependent degradation of a negative regulator of NAC1 [41]. The downregulation of NAC1 transcripts by either auxin-induced miR164 or ubiquitination may decrease auxin signals [42,43]. In this study, we observed that the lateral roots flourished more in drought-tolerant cultivar than in drought-susceptible cultivar (Figure 1B and Table 1); this might have resulted from the early accumulation of auxin responsive factors. In the early stage of dehydration stress, the drought-tolerant cultivar might change their morphological characteristics to enhance root and leaf development, thus accumulating more biomass to counteract the wastage brought on by dehydration stress.

miR166h is a member of the miR166 family and targets the Class III HD-ZIP protein 4 (HD-ZIP4 III) gene. In maize, miR166 family miRNAs cleave rolled leaf1 (rld1) mRNA which alters leaf polarity [44]. In addition to their involvement in leaf polarity regulation, HD-ZIP family members have been reported to be induced by various stress conditions, including drought and phytohormones [45,46]. Overexpression of the sunflower *Hahb-4* gene (a HD-ZIP gene) in *Arabidopsis* conferred both drought-resistance and morphological changes [47]. The class III HD-ZIP gene *AtHB8* is expressed in procambial tissues and has been functionally implicated in vascular tissue formation [48]. The class III HD-ZIP proteins have also been reported to control cambium activity by promoting axial cell elongation and xylem differentiation [49]. In this study, we found that the xylem and phloem cells of leaf are more in drought-tolerant cultivar than in drought-susceptible cultivar after dehydration treatment (Figure 1C and Table 2); this might have resulted from the upregulation of Class III HD-ZIP gene. In the course of dehydration stress, the drought-tolerant cultivar might regulate differentiation of vascular tissue cells, thus enhancing the developmental process to adapt dehydration stress.

Another miRNA, miR169d, is a member of the miR169 family and targets the CCAAT-box transcription factor (CCAAT-box TF), which is one of the most common elements in eukaryotic promoters. The nuclear factor Y (NFY) transcription factor complex was isolated as a CCAAT-binding protein complex and is an evolutionarily conserved transcription factor that occurs in a wide range of organisms, from yeast to human [50,51]. A study in *Triticum aestivum* revealed that nine subunits of the NFY complex were responsive to drought [52]. In *Arabidopsis*, transcription induced by drought and ABA was regulated by one NFY transcription factor (NFYA5), which might promote drought resistance [53]. In this study, miR169d was repressed in the drought-tolerant cultivar after dehydration stress, which might influence ABA-responsive transcription and result in enhanced dehydration stress tolerance.

The putative target of miR444d.3 is encoding a translation initiation factor 3 (IF3) gene. In eukaryotic protein synthesis, translational initiation is considered to be the rate-limiting step and controls transcript stability. IF3 plays a central role in polypeptide chain elongation in eukaryotes and its expression is induced by environmental stress [54,55]. Active conservation of polysomes during desiccation has been reported to be one of the mechanisms associated with stress tolerance in plants [56]. We found that miR444d.3 was downregulated in the drought-tolerant cultivar, indicating that IF3 may also involve in dehydration stress tolerance in wheat.

**Figure 7:**
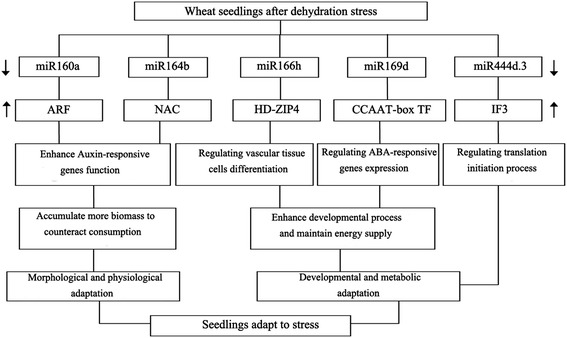
**Possible regulatory mechanism involving differentially expressed miRNAs and their target genes in two wheat genotypes under dehydration stress.** Different expression patterns of several miRNAs may be indirectly involved in wheat tolerance to dehydration stress by regulating target gene expression. ↑, upregulation; ↓, downregulation; ARF, auxin response factor; NAC, NAC transcription factor; HD-ZIP4, Class III HD-ZIP protein 4; CCAAT-box TF, CCAAT-box transcription factor; IF3, translation initiation factor 3.

We observed that growth of the drought-tolerant cultivar was better than that of the drought-susceptible cultivar after dehydration stress (Figure 1A and Additional file 1: Table S1). Given the high similarity in the genetic composition of the two genotypes, phenotypic variations—such as dehydration stress tolerance—are more likely to be caused by changes in regulatory processes than changes in proteins [57]. Because of their different geographical origins, the two genotypes are adapted to the particular environmental conditions in their native habitats. Thus, constitutive differences related to metabolism, biomass mobilization, energetic resources, radical system structure, and density of stomata would be expected. In this study, we confirmed that several miRNAs were downregulated in the drought-tolerant cultivar but upregulated in the drought-susceptible cultivar under dehydration stress, and we assessed the functions of their potential targets in response to stress. Therefore, we infer that the different capacities for dehydration stress tolerance in the two wheat genotypes may arise from the differential expression of target genes, which are regulated by their corresponding miRNAs (Figure 7).

## Conclusions

We found that 46 conserved miRNAs and 321 novel miRNAs were differentially expressed in two wheat genotypes under dehydration stress. Interestingly, 13 miRNAs exhibited opposite patterns of expression in the two wheat genotypes; these miRNAs were downregulated in drought-tolerant cultivar but upregulated in drought-susceptible cultivar. A number of representative miRNAs were verified by qPCR analysis and northern blot, which produced results consistent with those of the deep-sequencing method. Our findings indicate that expression patterns of some miRNAs may be very different even between two genotypes of the same species. Further analysis of the targets of differentially expressed miRNAs will help understand the mechanism of response and tolerance to dehydration stress in wheat.

## Methods

### Plant materials and treatments

Wheat cultivar Hanxuan10 and Zhengyin1 were used in this study. Hanxuan10 was collected from Luoyang Academy of Agriculture and Forestry Sciences, Luoyang City, Henan Province, China. Hanxuan10 is the important source in China with drought resistance, which is widely grown in semi-arid areas under rain-fed conditions. Zhengyin1 (St1472/506), which is generated from Akagomughi//Ritie/Wilhemina, was collected from the National Engineering Research Center for Wheat, Zhengzhou City, Henan Province, China. Seeds of Hanxuan10 and Zhengyin1 were surface-sterilized in 70% alcohol for 5 min, treated with 0.1% HgCl for 15 min, and rinsed five times in distilled water for 2 min each. After soaking in tap water for 12 h, the seeds were allowed to germinate for 4 days in a dark incubator at 25°C. The plantlets were then cultured in half strength Hoagland’s nutrient solution in a phytotron at 25°C/22°C (day/night) and under a 14-h photoperiod. Artificial water stress was induced with polyethylene glycol (PEG) 6000 solution to achieve an osmotic potential of −0.975 MPa (20% PEG). At the two-leaf stage, Hanxuan10 and Zhengyin1 seedlings were subjected to dehydration stress treatments designated T1 and T2, respectively, by watering with PEG solution or were grown under normal condition as control treatments designated C1 and C2, respectively. Leaf tissues were harvested from both sets of seedlings 12 h after treatment. All samples were frozen immediately in liquid nitrogen and stored at −80°C until use.

### Analysis of lateral roots, chlorophyll content and relative water content

Number and length of lateral root of the seedlings were recorded by counting and measurement. Chlorophyll in leaves was extracted with 80% acetone and its content was expressed as mg g^−1^ fresh weight (FW) as described previously [58]. Relative water content of leaf was calculated according to the method of Flexas et al. [59]. Data presented are the averages of at least 5 replicates, and the final data analysis used the t-test of Statistical Analysis System (SPSS 19.0) (SPSS Institute, Inc., NC, USA). In the results presented asterisks are used to identify the levels of significance: *P < 0.05 and **P < 0.01.

### Preparation and observation of leaf section

The fresh leaves of same position in C1, T1, C2 and T2 were used as materials, 0.5 × 0.5 cm tissues at the half zone of the leaf was taken, and these materials were fixed in FAA (Formalin: glacial acetic acid: 50% alcohol mixture = 5:5:90). Conventional paraffin section method [60] was used for making transverse section of every sample, safranin and fast green dyed and neutral gum sealing pieces. In the end, OLYMPUS BX51 microscope (Olympus Co., Japan) was used to observe the vascular tissue structure of the leaf and photograph. The observations were repeated three times per sample.

### Small RNA library construction and sequencing

Total RNA was extracted using TRIzol reagent (TaKaRa Co., Tokyo, Japan) according to the manufacturer’s instructions. Small RNAs were ligated sequentially to 5′ and 3′ RNA/DNA chimeric oligonucleotide adaptors (Illumina), and the resulting ligation products were gel purified by 10% denaturing PAGE and reverse-transcribed to produce cDNAs. The cDNAs were sequenced using a Genome Analyzer IIx System (Biomarker technologies CO., LTD, Beijing, China).

### Identification of miRNAs

The reads generated by deep sequencing were analyzed on the FASTX-toolkit website (http://hannonlab.cshl.edu/fastx_toolkit/). After the basic analysis, including filtering out low quality reads, trimming the adaptors and removing overrepresented sequences and noise, clean reads and unique reads (reads with non-redundancy) were obtained. The BlastN was used to align clean reads against Rfam 11.0 (ftp://ftp.sanger.ac.uk/pub/databases/Rfam) and Repbase (http://www.girinst.org/). The tRNA, rRNA, snoRNA and snRNA were annotated by aligning them to the Rfam database while the repeat sequences were aligned to the Repbase database. The remaining non-annotated sequences were used to do a BLAST against the miRBase 20 (http://www.mirbase.org) databases to identify mature miRNAs. All non-annotated reads with a length of 16–30 nt were mapped to the hexaploid wheat genome (http://www.cerealsdb.uk.net/CerealsDB/Documents/DOC_CerealsDB.php) using the Bowtie package (version one), only perfectly matched sRNAs were used for further analysis. Novel miRNAs were identified using the MIREAP [61] software (http://sourceforge.net/projects/mireap/) based on their precursors, followed by secondary structure prediction using RNAfold software (http://rna.tbi.univie.ac.at/cgi-bin/RNAfold.cgi). The key criteria for miRNA prediction were according to that had been reported in previous literature [20].

### Screening of differentially expressed miRNAs

Differentially expressed miRNAs were identified using the TPM and IDEG6 [62] software. TPM (Tags Per Million reads) is a standardized method for calculating miRNA expression levels. TPM values were calculated using the following equation:

$$ \mathrm{T}\mathrm{P}\mathrm{M}=\mathrm{number}\ \mathrm{of}\ \mathrm{mapped}\ \mathrm{miRNA}\ \mathrm{reads}/\mathrm{number}\ \mathrm{of}\ \mathrm{clean}\ \mathrm{sample}\ \mathrm{reads} \times {10}^6 $$

In order to calculate the levels of differential expressed miRNAs, normally the value was set as 0.01 by default when the sequencing read is 0 (no reads) [63]. We calibrated miRNA expression levels using multiple hypothesis tests with a false discovery rate (FDR) of less than 0.01, performed generalized chi-square tests for differential miRNA expression using the IDEG6 software (http://telethon.bio.unipd.it/bioinfo/IDEG6/), and screened the miRNAs for those with P-values less than 0.01 and TPM ratios between samples that were greater than 2 (fold change ≥ 2). The miRNAs that met these criteria were identified as being differentially expressed.

### Prediction of miRNA targets and annotation of functions

Potential miRNA targets were identified in wheat (*Triticum aestivum* L.) transcripts using the psRNATarget software (http://plantgrn.noble.org/psRNATarget/) (version 12) with the following parameters: prediction score cutoff value = 3.0, length for complementarity scoring = 20, and target accessibility = 25. Based on gene IDs, we obtained the sequences of miRNA targets from NCBI. Blast search, mapping, and annotation of these sequences were performed using the online software Blast2GO (http://www.blast2go.de).

### Reverse transcription reactions

Reverse transcription reactions were performed using an SYBR PrimeScript miRNA RT-PCR Kit (TaKaRa Co., Tokyo, Japan) following the manufacturer’s instructions. Briefly, a 20-μl reaction, containing 2-μl total RNA, 10-μl 2 × miRNA reaction buffer mix, 2-μl 0.1% BSA, and 2-μl miRNA PrimeScript RT enzyme mix was incubated at 37°C for 60 min and 85°C for 5 min and then stored at −20°C until use.

### Validation of differentially expressed miRNAs

qPCR was performed with a SYBR PrimeScript miRNA RT-PCR Kit (including reverse transcription and fluorescent quantitation) using a real-time PCR detection system (Bio-Rad laboratories, Inc.). Each 25-μl qPCR reaction solution comprised 2-μl cDNA (~100 ng), 1-μl 10 μM PCR forward primer, 1-μl 10 μM Uni-miR qPCR primer, 12.5-μl 2 × SYBR premix EX TaqII, and 8.5-μl nuclease-free water. The reactions were incubated at 95°C for 2 min and then subjected to 40 cycles of 95°C for 10 s, 58°C for 20 s, and 72°C for 10 s. After reactions were performed, a threshold was set manually and the threshold cycle (CT) was recorded automatically. All reactions were replicated three times per sample. The relative expression levels of the miRNAs were calculated using the 2^-ΔΔCT^ method [64], and the data were normalized to 18S rRNA CT values. The primer sequences corresponding to 25 differentially expressed miRNAs are presented in Additional file 6: Table S6.

### miRNA verification by northern blot

Northern blot analyses were performed with High Sensitive miRNA Northern Blot Assay Kit (Signosis, USA) in accordance with the manufacturer’s instructions. 30 μg total RNA of each sample was electrophoresed on a 15% polyacrylamide gel, transferred to membrane (Hybond N+ nylon filter, Amersham) with a semidry apparatus (BioRad, Hercules, CA) and UV crosslinked (Stratalinker; Stratagene). Membranes were exposed using a chemiluminescence imaging system (Ultralum, Inc., Claremont, CA). The normalization of the result was done by stripping the blot and probing it for U6 expression. Hybridization signals were imaged and quantified using a Molecular Image Analysis Software (Image Quant TL 7.0, GE Healthcare, USA).

### Validation of expression of the target genes by qPCR

The expression levels of the predicted target genes were estimated by qPCR. First strand cDNA was synthesized from 1 μg of RNA using a TransScript First-Strand cDNA Synthesis SuperMix (TransGen Co., Beijing, China) following the manufacturer’s instructions. The product of the reverse transcription reaction was diluted to a final volume of 90 μl, and 1 μl was used for qPCR with TransStart Top Green qPCR SuperMix (TransGen Co., Beijing, China). Each 20-μl qPCR reaction comprised 1-μl cDNA, 0.5-μl 10 μM forward primer, 0.5-μl 10 μM reverse primer, 10-μl 2 × TransStart Top Green qPCR SuperMix, and 8-μl double-distilled water. The reactions were incubated at 95°C for 2 min and then subjected to 40 cycles of 95°C for 5 s, 53°C for 20 s, and 72°C for 10 s. All reactions were replicated three times per sample. The relative expression level of the target gene was calculated using the 2^-ΔΔCT^ method normalized to 18s rRNA CT values. The sequences of the primer pairs used for the target genes are presented in Additional file 7: Table S7.

### Availability of supporting data

The generated raw reads of 4 small RNA libraries in this study are available in SNBI SRA database. The information can be found at the following links: http://www.ncbi.nlm.nih.gov/sra/?term=SRP051106. The accession numbers of C1, T1, C2 and T2 are SRX807431, SRX808858, SRX809318 and SRX809338, respectively. The data including the chlorophyll content and relative water content are available in Additional file 1. The sequences of differentially expressed conserved miRNAs and novel miRNAs are available in Additional files 2 and 3, respectively. Secondary structure of differentially expressed novel miRNAs is available in Additional file 4. Potential target genes of differentially expressed miRNAs are available in Additional file 5. All primer sequences used in this study are listed in Additional files 6 and 7, respectively.

## Additional files

Additional file 1: Table S1. Changes in the content of chlorophyll and relative water content in two wheat genotypes under dehydration stress. Additional file 2: Table S2. Differentially expressed conserved miRNAs in two wheat genotypes after dehydration stress. Additional file 3: Table S3. Differentially expressed novel miRNAs in two wheat genotypes after dehydration stress. Additional file 4: Table S4. Secondary structure of differentially expressed novel miRNAs. Additional file 5: Table S5. Potential target genes of differentially expressed miRNAs. Additional file 6: Table S6. Primer sequences used for qPCR analysis of 25 differentially expressed miRNAs. Additional file 7: Table S7. Primer pair sequences used for qPCR analysis of 10 target genes.
